# Acridine Based Small Molecular Hole Transport Type Materials for Phosphorescent OLED Application

**DOI:** 10.3390/molecules26247680

**Published:** 2021-12-19

**Authors:** Ramanaskanda Braveenth, Keunhwa Kim, Il-Ji Bae, Kanthasamy Raagulan, Bo Mi Kim, Miyoung Kim, Kyu Yun Chai

**Affiliations:** 1 Division of Bio-Nanochemistry, College of Natural Sciences, Wonkwang University, Iksan 570-749, Jeonbuk, Korea; braveenth.czbt@gmail.com (R.B.); qeutesl@naver.com (K.K.); raagulan@live.com (K.R.); 2 Nano-Convergence Research Center, Korea Electronics Technology Institute, Jeonju 54853, Jeonbuk, Korea; ijbae@keti.re.kr; 3 Department of Chemical Engineering, Wonkwang University, Iksan 570-749, Jeonbuk, Korea; 123456@wku.ac.kr

**Keywords:** organic light emitting diodes, HTL, host material, acridine, triphenylamine, carbazole

## Abstract

Two small molecular hole-transporting type materials, namely 4-(9,9-dimethylacridin-10(9*H*)-yl)-*N*-(4-(9,9-dimethylacridin-10(9*H*)-yl)phenyl)-*N*-phenylaniline (TPA-2ACR) and 10,10′-(9-phenyl-9*H*-carbazole-3,6-diyl)bis(9,9-dimethyl-9,10-dihydroacridine) (PhCAR-2ACR), were designed and synthesized using a single-step Buchwald–Hartwig amination between the dimethyl acridine and triphenylamine or carbazole moieties. Both materials showed high thermal decomposition temperatures of 402 and 422 °C at 5% weight reduction for PhCAR-2ACR and TPA-2ACR, respectively. TPA-2ACR as hole-transporting material exhibited excellent current, power, and external quantum efficiencies of 55.74 cd/A, 29.28 lm/W and 21.59%, respectively. The achieved device efficiencies are much better than that of the referenced similar, 1,1-Bis[(di-4-tolylamino)phenyl]cyclohexane (TAPC)-based device (32.53 cd/A, 18.58 lm/W and 10.6%). Moreover, phenyl carbazole-based PhCAR-2ACR showed good device characteristics when applied for host material in phosphorescent OLEDs.

## 1. Introduction

Organic light emitting diodes (OLEDs) have drawn attention from research and commercial communities due to their advantages, such as wide view angle, thin films, high brightness, high contrast and less power consumption compared to conventional displays. Interestingly, OLED technology revealed the possibility of achieving 100% internal quantum efficiencies (IQE) by utilizing second-generation phosphorescence and third-generation thermally activated delayed fluorescence dopants [1,2,3,4]. At the same time, OLED technology showed immiscible progress in material and device development. Moreover, multilayer OLED devices revealed efficiency enhancement compared to conventional display devices. In general, multilayer OLED device structure consists of an electron transporting layer (ETL), an electron injecting layer (EIL), an electron blocking layer (EBL), a hole-transporting layer (HTL), a hole injecting layer (HIL), and an emission layer (EML), and all layers are sandwiched between the anode and the cathode [5,6,7,8]. 

When compared to electron carriers, hole-transporting materials (HTM) play a key role in hole injection and transportation to support the charge balance at the emission layer. An efficiency device requires choice of a suitable HTM to obtain balance carriers. As basic requirements, HTM should possess high hole injection ability and hole mobility, an appropriate frontier molecular orbital energy level, high ionization potential and thermal stability [9,10,11,12]. Generally, hole-transporting materials show an electron donating nature, so the chemical skeleton composed of diphenylamine, carbazole, spirobifluorene derivatives are used widely in practical applications. The most commonly used HTMs are 1,1-Bis[(di-4-tolylamino)phenyl]cyclohexane (TAPC), 2,7-Bis[*N*-(1-naphthyl)anilino]-9,9′-spirobi [9*H*-fluorene] (Spiro-NPB), *N*,*N*′-Di(1-naphthyl)-*N*,*N*′-diphenyl-(1,1′-biphenyl)-4,4′-diamine (NPB), and *N*,*N*′-Bis(3methylphenyl)-*N*,*N*′-diphenylbenzidine (TPD). This type of well-known materials grab solid attention in the development of small molecular type HTMs with certain modification in their molecular skeleton [13,14,15].

Host materials are known for energy-supplying elements in the emission layer, and they should have higher triplet energy than that of the dopant to prevent the reverse energy flow back from the dopant and host materials. Moreover, host materials help to suppress the triplet–triplet annihilation (TTA) and aggregation-caused quenching (ACQ) in case of phosphorescent OLEDs. There are three types of host materials reported according to their molecular structure, which are hole-transporting type (HT), electron transporting type (ET) and bipolar type host materials. A desirable host material should have adequate carrier mobility, an appropriate highest occupied molecular orbital (HOMO) and lowest unoccupied molecular orbital (LUMO) energy level to reduce the energy barrier to allow smooth carrier transportation. Carbazole derivatives are a well-known backbone in host applications due to their high triplet energy (3 eV), and 4,4′-Bis(*N*-carbazolyl)-1,1′-biphenyl (CBP) is one of the widely used carbazole-based HT type host materials in phosphorescent OLED applications [16,17,18,19,20,21,22,23].

**Scheme 1:**
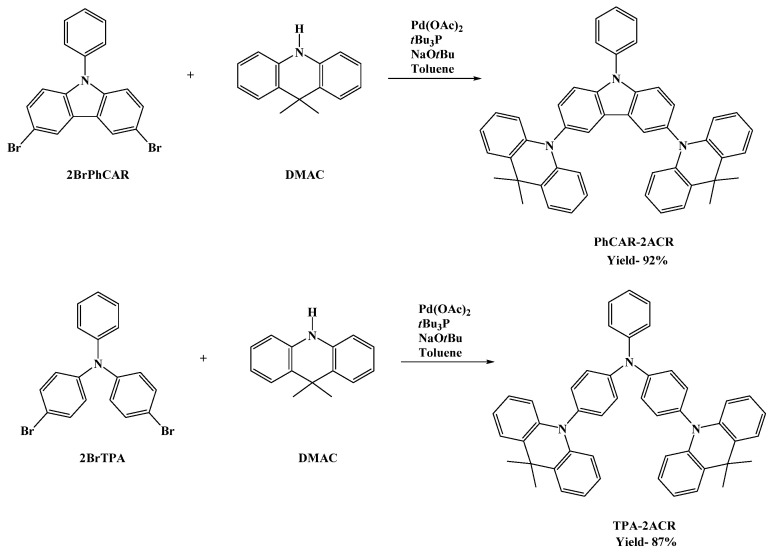
Synthetic route of target molecules of PhCAR-2ACR and TPA-2ACR.

In this work, we have designed and synthesized dimethyl acridine-substituted triphenylamine and carbazole core-based 4-(9,9-dimethylacridin-10(9*H*)-yl)-*N*-(4-(9,9-dimethylacridin-10(9*H*)-yl)phenyl)-*N*-phenylaniline (TPA-2ACR) and 10,10′-(9-phenyl-9*H*-carbazole-3,6-diyl)bis(9,9-dimethyl-9,10-dihydroacridine) (PhCAR-2ACR), respectively, for their application in hole-transporting layer and host materials for yellow phosphorescent OLEDs (Scheme 1). Here, we have analysed the photophysical properties and device performances with reference molecules. Triphenylamine based TPA-2ACR showed better performance as a hole-transporting material, while carbazole-based Ph-CAR-2ACR revealed the good characteristics of the HT type host material.

## 2. Results

**Figure 1:**
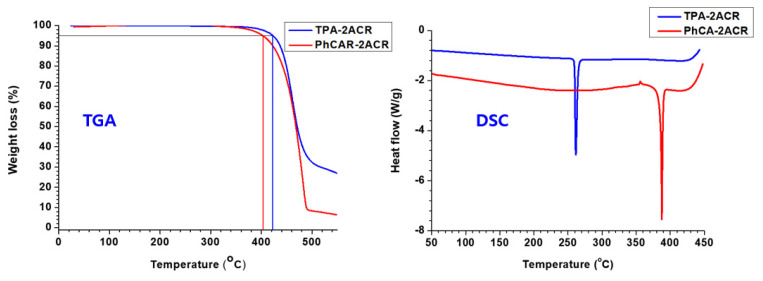
Thermal gravimetric analysis (TGA) and differential scanning calorimetry (DSC) of TPA-2ACR and PhCAR-2ACR.

The thermal properties were analysed by using thermal gravimetric analysis (TGA) and differential scanning calorimetry (DSC). TPA-2ACR and PhCAR-2ACR exhibited thermal decomposition temperatures of 422 and 402 °C at 5% weight reduction. The melting points were recorded as 261 and 387 °C, respectively (Figure 1). We believe that above thermal stabilities are enough to support the morphological and film forming ability during the device fabrication.

**Figure 2:**
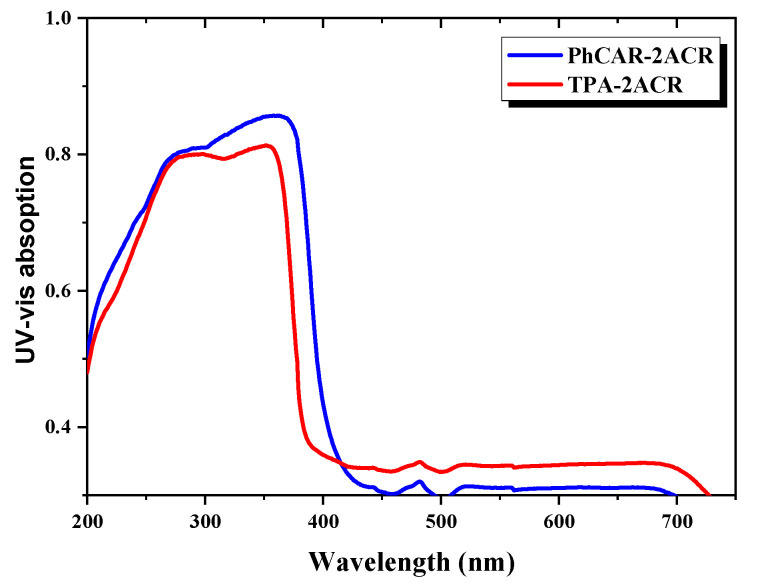
UV-vis absorption (in toluene) spectra of PhCAR-2ACR and TPA-2ACR.

Both molecules showed prominent absorption around 277 nm, and further absorptions observed at 351 and 359 nm for TPA-2ACR and PhCAR-2ACR, respectively (Figure 2). The optical band gap energy values were calculated from the onset absorption wavelengths of 430 and 420 nm, and the calculated values were 2.89 and 2.96 eV for PhCAR-2ACR and TPA-2ACR, respectively. 

**Figure 3:**
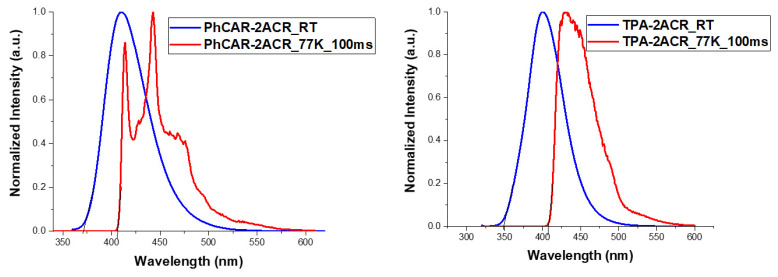
Room temperature and low temperature (100 ms) photoluminescence (PL) spectra of PhCAR-2ACR and TPA-2ACR.

The photoluminescence spectra of both materials were recorded at room and low temperature in toluene solution (Figure 3 and Table 1). The room temperature PL of PhCAR-2ACR and TPA-2ACR were observed at 403 and 401 nm. The singlet energy levels were known from the onset wavelengths of room temperature PL, which were 3.35 and 3.55 eV, while the triplet energy values of 3.05 and 3.04 eV were obtained from the low-temperature spectra (onset wavelength) for PhCAR-2ACR and TPA-2ACR, respectively. We noticed that both materials showed higher triplet energy, which can help to prevent the triplet quenching when it applies to the HTM and host. At the same time, we anticipated that our materials as host application would be able to supply energy to the dopant without any energy flow back from the dopant. Thus, this type of molecule can express multifunctional properties in phosphorescent OLED applications. 

**Figure 4:**
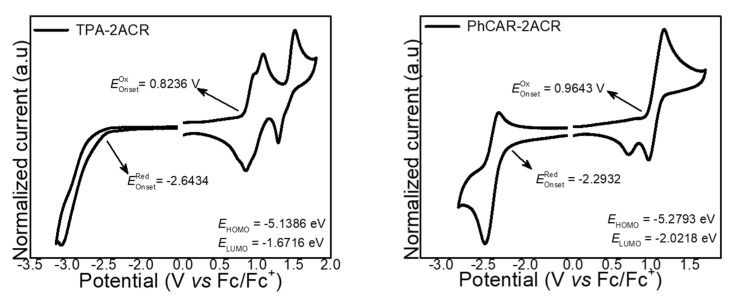
Cyclic voltammetry measurements of TPA-2ACR and PhCAR-2ACR.

The electrochemical studies were carried out using cyclic voltammetry measurements (Figure 4). The HOMO and LUMO energy levels of synthesized materials were measured from the onset values of oxidation and reduction potential in cyclic voltammetry with a scanning rate of 100 mV/S using an Ag wire in 0.01 M AgNO_3_ as a counter and 0.1 M tetrabutyl ammonium perchlorate as working electrodes and using an internal ferrocene/ferrocenium (Fc/Fc+) as a standard (E1/2(ferrocene) is equal to 0.493 eV). The HOMO energy level of PhCAR-2ACR and TPA-2ACR were calculated from the oxidation potential using the following Equation (1) (E_HOMO_—HOMO energy level; E_ox_—Oxidation potential; E_1/2_(_ferrocene_—half wave potential of ferrocene):

E_HOMO_ = [(E_ox_ − E_1/2(ferrocene)_) + 4.8] eV, (1)

The calculated HOMO energy values were 5.27 and 5.13 eV for PhCAR-2ACR and TPA-2ACR, respectively. The LUMO energy levels were obtained from the reduction potential using the following Equation (2) (E_LUMO_—LUMO energy level; E_red_—reduction potential):

E_LUMO_ = [(E_red_ − E_1/2(ferrocene)_) + 4.8] eV, (2)

The LUMO energy levels were 2.02 and 1.67 eV for PhCAR-2ACR and TPA-2ACR, respectively. TPA-2ACR exhibited a higher HOMO energy level when compared to carbazole-based PhCAR-2ACR materials, so we can expect that triphenylamine-based TPA-2ACR will show good hole-transporting ability in OLED devices.

**Figure 5:**
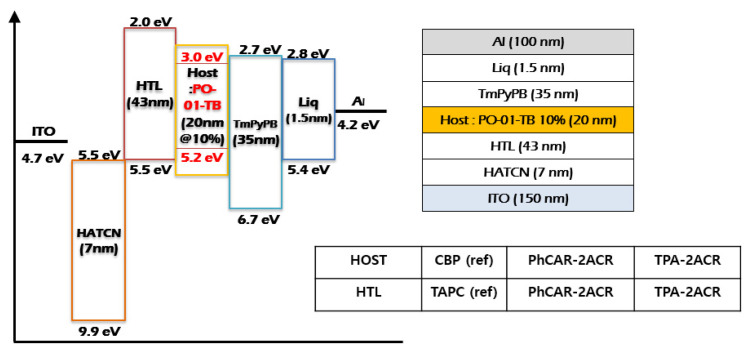
Device energy diagram and device structure of phosphorescent OLEDs.

To understand the hole-transporting properties of synthesized materials, phosphorescent OLED devices were fabricated, and the fabrication procedures are explained in Section 3.5 (Figure 5). TAPC was used as reference HTM to compare the device properties with our synthesized materials.

The current efficiency of TAPC, PhCAR-2ACR and TPA-2ACR-based yellow phosphorescent OLEDs were 32.53, 23.13 and 55.74 cd/A, respectively (Figures S1 and S2 and Table 2). The power efficiencies were 18.58, 6.05 and 29.28 lm/W for TAPC, PhCAR-2ACR and TPA-2ACR, respectively. Triphenylamine-based TPA-2ACR showed excellent current and power efficiencies when compared to reference TAPC and carbazole-based PhCAR-2ACR. The external quantum efficiencies (EQEs) of TAPC, PhCAR-2ACR and TPA-2ACR based devices were recorded as 10.6, 7.65 and 21.59%, respectively. Triphenylamine-based TPA-2ACR revealed two-time higher EQE than reference TAPC-based similar device. Since carbazole is a weak donor, PhCAR-2ACR showed comparatively lower device efficiencies. Moreover, triphenylamine has a strong electron-donating moiety when compared to carbazole moiety, so better device performance of TPA-2ACR can be attributed to its superior hole-transporting ability. 

**Figure 6:**
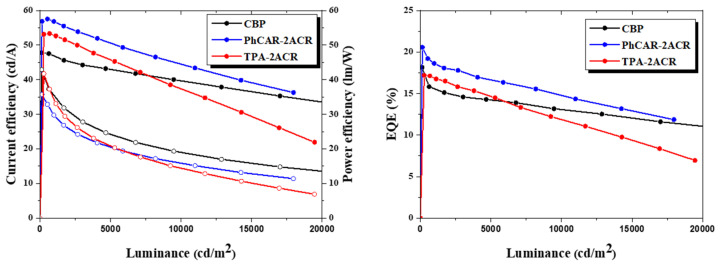
Luminescence vs. current and power efficiency, and luminance vs. external quantum efficiencies of yellow phosphorescent OLEDs with CBP, PhCAR-2ACR and TPA-2ACR as host materials.

To investigate the properties of synthesized materials further as a host application, we have fabricated another set of yellow phosphorescent OLEDs using PhCAR-2ACR and TPA-2ACR as hole-transporting (HT)-type host materials. CBP host material was used as the reference HT-type host material for our current study. The current efficiencies of CBP, PhCAR-2ACR and TPA-2ACR were 47.83, 56.90 and 53.15 cd/A and power efficiencies were 42.94, 35.75 and 41.74 lm/W, respectively (Figure 6). The maximum EQEs of CBP, PhCAR-2ACR and TPA-2ACR-based devices were 18.16, 20.57 and 17.20%, respectively. Carbazole-based PhCAR-2ACR exhibited excellent current and power efficiencies. We believe that the high triplet energy of carbazole moiety supports an effective energy transfer to the yellow dopant while preventing the reverse energy flow back to the host. 

**Figure 7:**
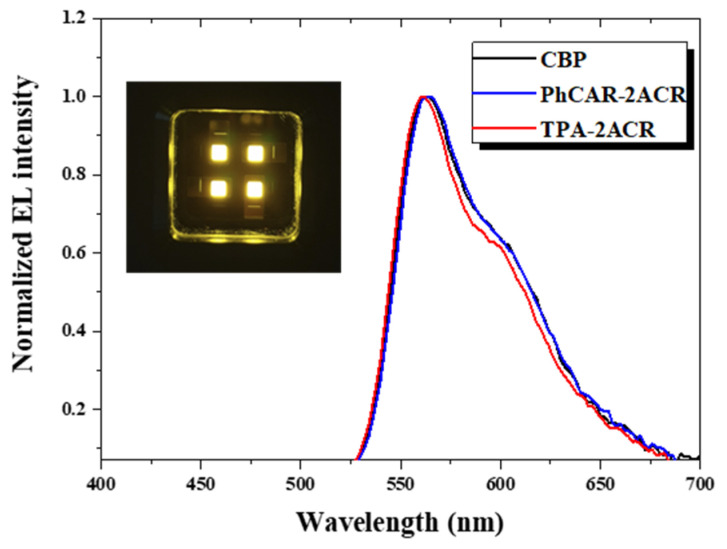
Electroluminescent spectra of yellow phosphorescent OLEDs.

Our synthesized materials, especially TPA-2ACR, showed good device performance as a hole transporter and host in yellow phosphorescence OLEDs. These types of materials are very useful in OLED applications due to their versatile capability of HTM as well as the host. Additionally, carbazole-based PhCAR-2ACR showed poor performance as hole-transporting material, but it revealed an excellent performance as the host due to its high triplet energy level. The electroluminescence spectra showed emission maxima around 560 nm, and it resembled the emission of the yellow dopant PO:O1 (Figure 7). From our results, we can understand that there is no prominent shift in the observed emission spectra.

## 3. Materials and Methods

### 3.1. Materials

3,6-dibromo-9-phenyl-9*H*-carbazole (2BrPhCAR), 4-bromo-*N*-(4-bromophenyl)-*N*-phenylaniline (2BrTPA) and 9,9-dimethyl-9,10-dihydroacridine (DMAC) were purchased from TCI chemicals (Seoul, Korea). Pd(OAc)_2_, *t*Bu_3_P, NaO*t*Bu and anhydrous toluene were obtained from Sigma-Aldrich (Seoul, Korea). Dichloromethane and *n*-hexane were bought from SK chemicals (Seongnam-si, Gyeonggi-do, Korea). Analytical thin-layer chromatography (TLC) was carried out using Merck Kieselgel 60-coated TLC plate with visualizations of 254 and 365 nm (Seoul, Korea). 

### 3.2. Instrumentation

^1^H and ^13^C NMR characterization were performed with a JEON JNM-ECP FT-NMR spectrometer (Peabody, MA, USA) operating at 500 MHz (Figures S3–S6). Photoluminescence (PL) spectra were recorded with a Jasco FP-8300 spectrophotometer. (Easton, MD, USA). Mass spectrometry analysis measured with a Xevo TQ-S spectrometer (Waters, Milford, MA, USA). Thermal gravimetric analysis (TGA) and differential scanning calorimetry (DSC) were measured with a PerkinElmer DSC 4000 and TGA 8000 system (Melville, NY, USA) under a nitrogen atmosphere with a heating rate of 10 °C/min. The triplet energy level (ET) was calculated from the onset wavelength of the emission spectra at 77 K in 10^−5^ M toluene. The HOMO (highest occupied molecular orbital) and LUMO (lowest unoccupied molecular orbital) values were determined by cyclic voltammetry. OLED devices were fabricated with a thermal evaporating system under a pressure of 5 × 10^−7^ torr (Sunicel plus, Seoul, Korea). Current density–voltage–luminescence (*J–V–L*) performances were recorded by an OLED *I–V–L* test system (Polarmix M6100, Suwon, Korea). The electroluminescence (EL) spectra were measured with a spectroradiometer (Konica Minolta CS-2000, Tokyo, Japan). 

### 3.3. Synthesis and Characterization of 10,10′-(9-Phenyl-9H-carbazole-3,6-diyl)bis(9,9-dimethyl-9,10-dihydroacridine) PhCAR-2ACR

A mixture of 3,6-dibromo-9-phenyl-9*H*-carbazole 2BrPhCAR (2.0 g, 4.9 mmol), 9,9-dimethyl-9,10-dihydroacridine DMAC (2.29 g, 10.9 mmol), palladium (II) acetate (0.05 g, 0.2 mmol), tri-tert-butylphosphine (0.08 g, 0.4 mmol) and sodium tert-butoxide (0.2 g, 1.9 mmol) were added in a two-neck round-bottom flask equipped with a condenser. Anhydrous toluene of 30 mL added, then subjected to inert condition by using nitrogen gas. The mixture stirred at 110 °C for 8 h under inert condition, and reaction progress was observed using thin layer chromatography. After completion of the reaction, reaction mixture was filtered through silica plug and worked up using water and dichloromethane several times. The organic layer was dried under anhydrous magnesium sulphate and concentrated under reduced pressure. Crude mixture was recrystallized from dichloromethane and *n*-Hexane to obtain the pure target material PhCAR-2ACR. 

Yield: 92%; white solid; ^1^H NMR (500 MHz, CDCl_3_) δ 8.07 (s, 2 H), 7.67–7.75 (m, 6 H), 7.57 (t, *J* = 7 Hz, 1 H), 7.45–7.47 (m, 4 H), 7.39 (d, *J* = 9 Hz, 2 H), 6.89–6.96 (m, 8 H), 6.36 (d, *J* = 8 Hz, 4 H), 1.71 (s, 12 H); ^13^C NMR (125 MHz, CDCl_3_) δ 141.6, 140.8, 137.3, 133.8, 130.2, 130.1, 129.5, 128.2, 127.2, 126.3, 125.2, 125.0, 123.4, 120.4, 114.3, 112.3, 31.3; MS (APCI) *m*/*z*: [(M + H)^+^] 658.33 for C_48_H_39_N_3_; (HRMS) MALDI-TOF: found [(M + H)^+^] 657.3132 molecular formula C_48_H_39_N_3_ requires [(M + H)^+^] 657.3144.

### 3.4. Synthesis and Characterization of 4-(9,9-Dimethylacridin-10(9H)-yl)-N-(4-(9,9-dimethylacridin-10(9H)-yl)phenyl)-N-phenylaniline TPA-2ACR

A mixture of 4-bromo-*N*-(4-bromophenyl)-*N*-phenylaniline 2BrTPA (2.0 g, 4.9 mmol), 9,9-dimethyl-9,10-dihydroacridine DMAC (2.29 g, 10.9 mmol), palladium (II) acetate (0.05 g, 0.2 mmol), tri-tert-butylphosphine (0.08 g, 0.4 mmol) and sodium tert-butoxide (0.2 g, 1.9 mmol) were added in a two-neck round-bottom flask equipped with condenser. Anhydrous toluene of 40 mL added, then subjected to inert conditions using nitrogen gas. The mixture stirred at 110 °C for 8 h under inert conditions, and reaction progress was observed using thin-layer chromatography. After completion of the reaction, the reaction mixture was filtered through a silica plug and worked up using water and chloroform several times. The organic layer was dried under anhydrous magnesium sulphate and concentrated under reduced pressure. Crude mixture was recrystallized from *n*-Hexane to obtain the pure target material TPA-2ACR.

Yield: 87%; white solid; ^1^H NMR (500 MHz, CDCl_3_) δ 7.46 (d, *J* = 6 Hz, 4 H), 7.40–7.43 (m, 6 H), 7.34 (d, *J* = 7.5 Hz, 2 H), 7.24–7.25 (m, 4 H), 7.17 (t, *J* = 7 Hz, 1 H), 7.02–7.05 (m, 4 H), 6.93–9.96 (m, 4 H), 6.43 (d, *J* = 7 Hz, 4 H), 1.69 (s, 12 H); ^13^C NMR (125 MHz, CDCl_3_) δ 147.3, 141.1, 135.4, 132.1, 130.1, 129.8, 126.4, 125.5, 125.2, 124.2, 120.6, 114.1, 31.3; MS (APCI) *m*/*z*: 660.22 for C_48_H_41_N_3_ [(M + H)^+^]; (HRMS) MALDI-TOF: Found [(M + H)^+^] 659.3294 molecular formula C_48_H_41_N_3_ requires [(M + H)^+^] 659.3300.

### 3.5. Device Fabrication 

The OLED device fabrication process is as follows: indium–tin–oxide (ITO)-coated glass substrates were cleaned and washed in isopropyl alcohol ultrasonic bath, and further washing conducted with deionized water for 20 min. Then, the cleaned substrates underwent UV–ozone treatment for about 10 min. All the organic layers along with the metal cathode were fabricated on the pre-washed ITO coated glass substrate. The deposition rate of layers was applied around 5 × 10^−7^ Torr using a Sunic organic evaporator inside a glove box under inert conditions. The fabricated device structure was as follows: indium tin oxide ITO (150 nm)/1,4,5,8,9,11-Hexaazatriphenylenehexacarbonitrile HATCN (7 nm)/HTL (43 nm)/4,4′-Bis(*N*-carbazolyl)-1,1′-biphenyl CBP:10% Iridium(III) bis(4-(4-tert-butylphenyl) thieno[3,2-c]pyridinato-*N*,C2′) acetylacetonate PO-01 (20 nm)/1,3,5-Tri(m-pyridin-3-ylphenyl)benzene TmPyPB (35 nm)/8-Quinolinolato lithium Liq (1.5 nm)/Aluminum Al (100 nm). ITO and Al were used as anode and cathode, respectively. HATCN used as hole injecting layer (HIL), Liq applied as electron injecting layer (EIL) and TmPyPB used as electron transport layer (ETL). Yellow phosphorescent dopant of PO-O1 used for all devices in this study.

## 4. Conclusions

In this work, we designed and synthesized two small molecular hole-transporting-type materials. TPA-2ACR and PhCAR-2ACR were designed and synthesized between dimethyl acridine and triphenylamine or carbazole core. We utilized our synthesized materials for hole transporting and host materials. TPA-2ACR as a hole-transporting material exhibited excellent current, power, and external quantum efficiencies of 55.74 cd/A, 29.28 lm/W, and 21.59%, respectively when compared to that of the referenced similar TAPC-based device (32.53 cd/A, 18.58 lm/W, and 10.6%). The triplet energy of our materials was more than 3 eV, and they both showed excellent device performances. The phenyl carbazole-based PhCAR-2ACR showed current and external quantum efficiencies of 56.90 cd/A and 20.57%, respectively and the referenced CBP-based device revealed 47.83 cd/A and 18.16%. We believe that our acridine-based small molecular hole transport type materials can replace the TAPC hole-transporting and CBP host material in future OLED applications.

## Figures and Tables

**Table 1:** Thermal, photophysical and electrochemical properties of PhCAR-2ACR and TPA-2ACR.

Materials	PhCAR-2ACR	TPA-2ACR
T*_d_* (°C)	402	422
PL (nm)	403	401
Band gap (eV)	2.89	2.96
Triplet energy (eV)	3.05	3.04
HOMO (eV)	5.27	5.13
LUMO (eV)	2.02	1.67

**Table 2:** Device characteristics of PhCAR-2ACR and TPA-2ACR-based phosphorescent devices with TAPC and CBP reference devices.

Device Characteristics	PhCAR- 2ACR (HTL)	TPA- 2ACR (HTL)	TAPC (Reference)	PhCAR- 2ACR (Host)	TPA- 2ACR (Host)	CBP (Reference)
Current Efficiency (cd/A)	23.13	55.74	32.53	56.90	53.15	47.83
Power Efficiency (lm/W)	6.05	29.28	18.58	35.75	41.74	42.94
EQE (%)	7.65	21.59	10.6	20.57	17.20	18.16
CIE (x, y)	0.49, 0.49	0.49, 0.49	0.49, 0.49	0.50, 0.50	0.49, 0.51	0.50, 0.50

## Data Availability

Not applicable.

## Supplementary Materials

The following are available online, Figure S1: Luminescence-current and power efficiency, and luminance vs external quantum efficiencies of yellow phosphorescent OLEDs with TAPC, PhCAR-2ACR and TPA-2ACR as hole transporting materials (HTMs), Figure S2: Luminescence-current density vs voltage of host and HTL based yellow phosphorescent OLEDs devices (TAPC, PhCAR-2ACR and TPA-2ACR), Figure S3: Proton NMR of PhCAR-2ACR, Figure S4: Carbon NMR of PhCAR-2ACR, Figure S5: Proton NMR of TPA-2ACR, Figure S6: Carbon NMR of TPA-2ACR.

## Sample Availability

Samples of the compounds are available from the authors.
