# Supplementary material for: Acridine Based Small Molecular Hole Transport Type Materials for Phosphorescent OLED Application
Source: Molecules. 2021 Dec 19;26(24):7680. doi: 10.3390/molecules26247680 (PMC8703516; doi:10.3390/molecules26247680)
Supplement: Supplementary file 1 [file molecules-26-07680-s001.zip › molecules-1430673-supplementary.pdf]

Supporting information

Acridine Based Small Molecular Hole Transport Type Materials for Phosphorescent OLED Application

**Ramanaskanda Braveenth [1], Keunhwa Kim [1], Il-Ji Bae [2], Kanthasamy Raagulan [1], Bo Mi Kim [3], Miyoung Kim [2,*]
and Kyu Yun Chai [1,*]**

[1] Division of Bio-Nanochemistry, College of Natural Sciences, Wonkwang University, Iksan City, Chonbuk 570-749, Korea; braveenth.czbt@gmail.com (R.B.); qeutesl@naver.com (K.K.); raagulan@live.com (K.R.)

[2] Nano-Convergence Research Center, Korea Electronics Technology Institute, Jeonju 54853, Korea; ijbae@keti.re.kr

[3] Department of Chemical Engineering, Wonkwang University, Iksan City, Chonbuk 570-749, Korea; 123456@wku.ac.kr

* Correspondence: miy1kim@keti.re.kr (M.K.); geuyoon@wonkwang.ac.kr (K.Y.C.); Tel.: +82-632-190-011 (M.K.); +82-638-506-230 (K.Y.C.)

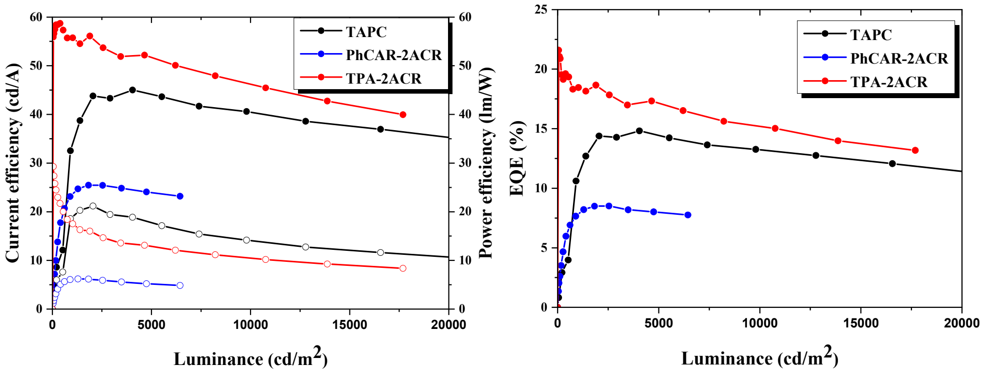

Figure S1. Luminescence-current and power efficiency, and luminance vs external quantum efficiencies of yellow phosphorescent OLEDs with **TAPC**, **PhCAR-2ACR** and **TPA-2ACR** as hole transporting materials (HTMs).

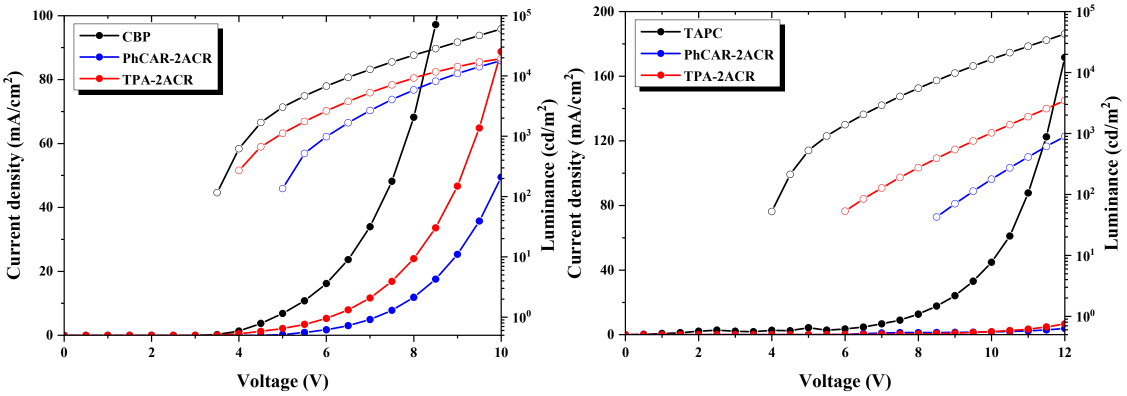

Figure S2. Luminescence-current density vs voltage of host and HTL based yellow phosphorescent OLEDs devices (**TAPC**, **PhCAR-2ACR** and **TPA-2ACR**).

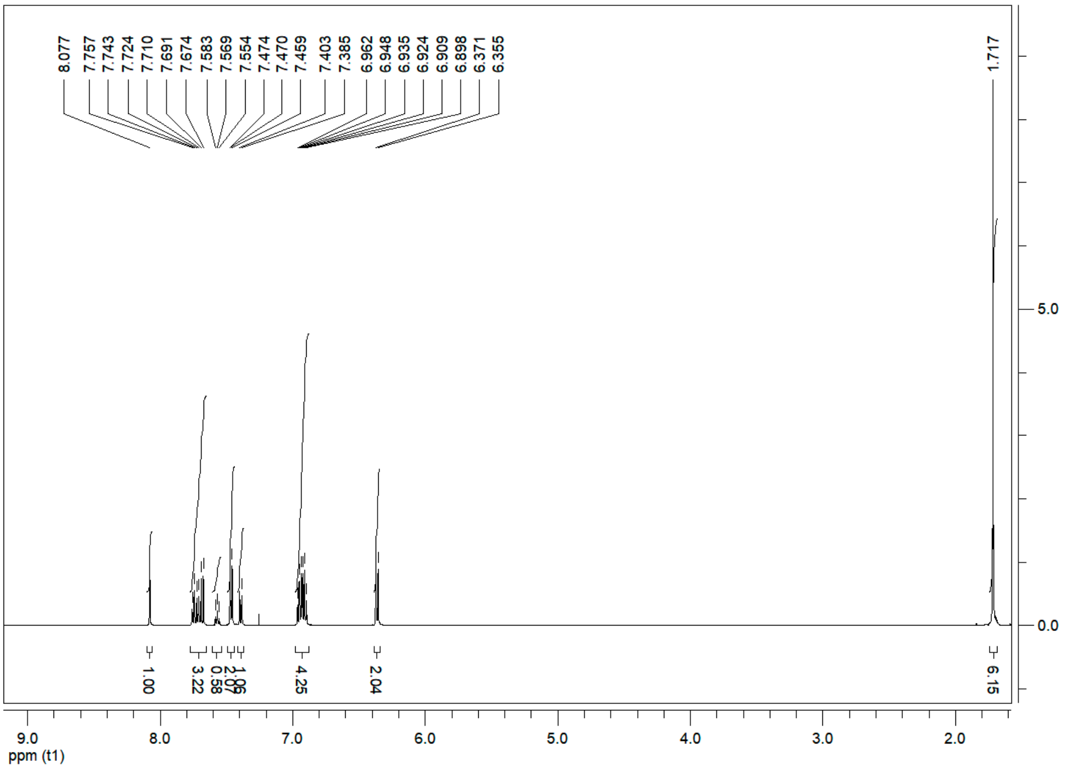

Figure S3. Proton NMR of **PhCAR-2ACR**.

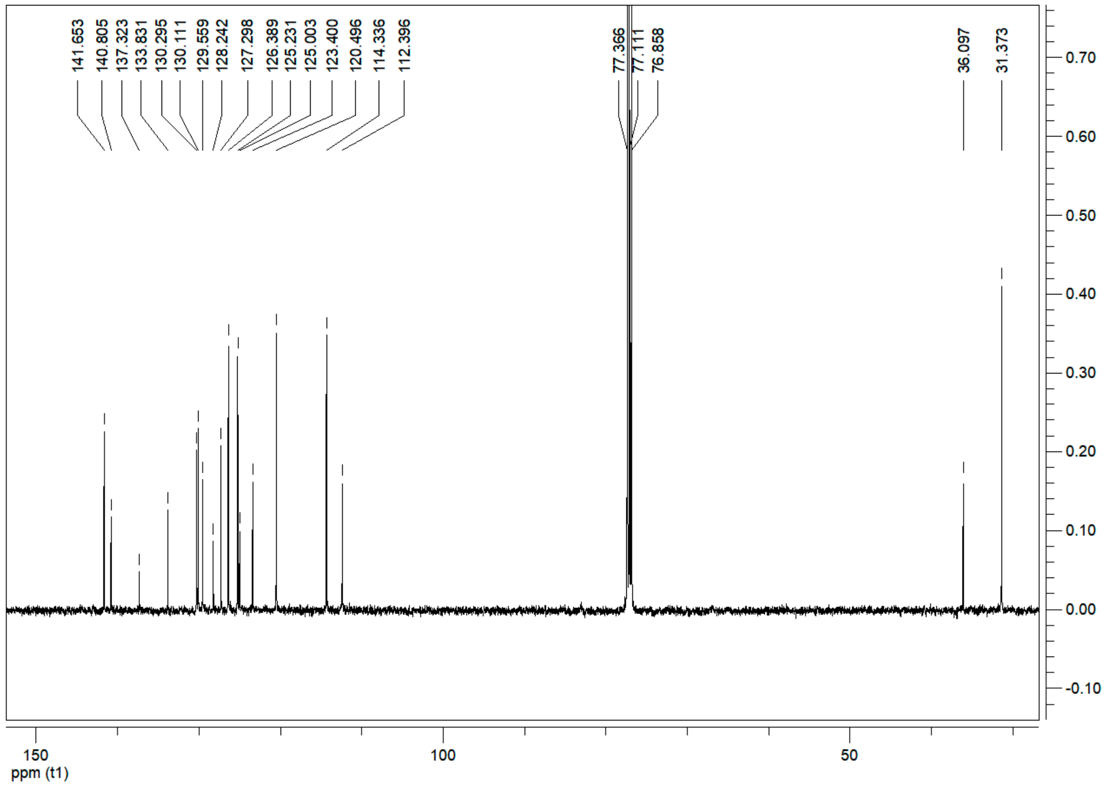

Figure S4. Carbon NMR of **PhCAR-2ACR**.

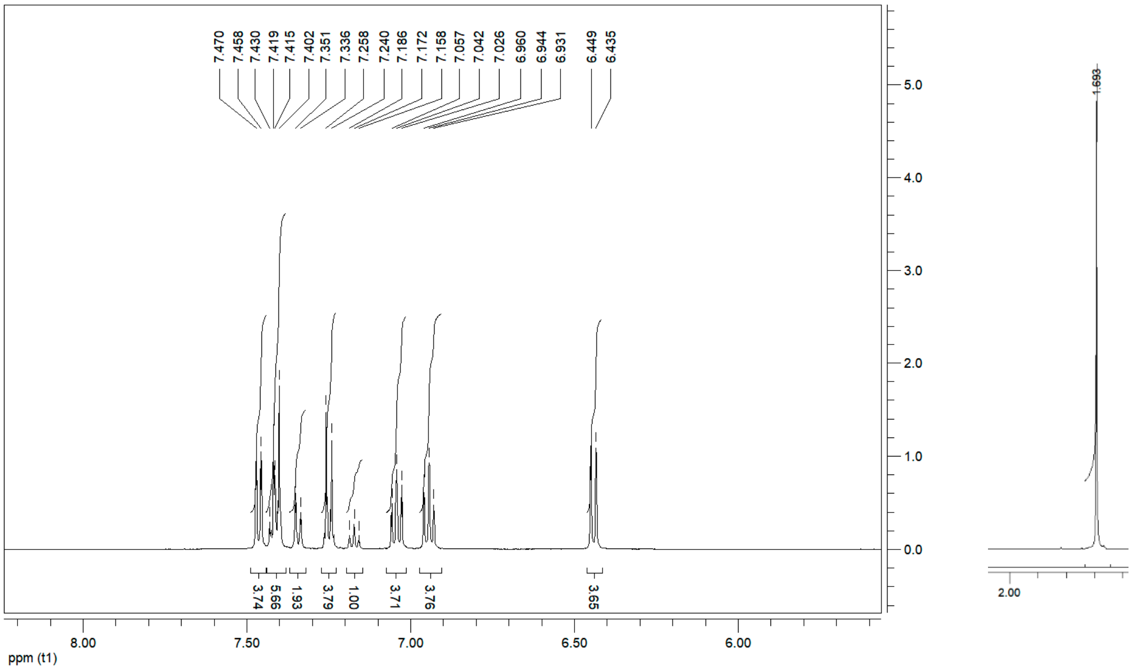

Figure S5. Proton NMR of **TPA-2ACR**.

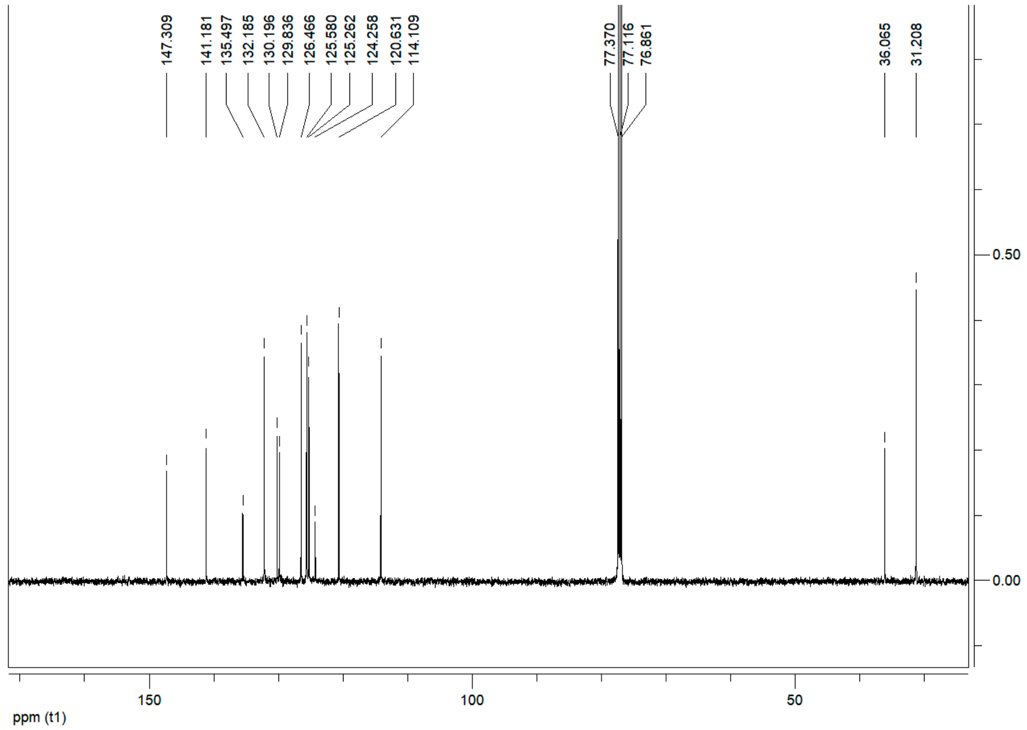

Figure S6. Carbon NMR of **TPA-2ACR**.
